# Through the looking glass: the neural basis of self-concept in young adults with antisocial trajectories

**DOI:** 10.1093/scan/nsad016

**Published:** 2023-03-17

**Authors:** Ilse H van de Groep, Marieke G N Bos, Lucres M C Jansen, Arne Popma, Eveline A Crone

**Affiliations:** Erasmus School of Social and Behavioural Sciences, Erasmus University, Rotterdam 3062 PA, The Netherlands; Leiden Institute for Brain and Cognition, Leiden University, Leiden 2300 RC, The Netherlands; Department of Child and Adolescent Psychiatry, Amsterdam University Medical Center, 1100 DD Amsterdam Zuidoost, The Netherlands; Developmental and Educational Psychology, Faculty of Social and Behavioural Sciences, Leiden University, Leiden 2333 AK, The Netherlands; Leiden Institute for Brain and Cognition, Leiden University, Leiden 2300 RC, The Netherlands; Developmental and Educational Psychology, Faculty of Social and Behavioural Sciences, Leiden University, Leiden 2333 AK, The Netherlands; Department of Child and Adolescent Psychiatry, Amsterdam University Medical Center, 1100 DD Amsterdam Zuidoost, The Netherlands; Department of Child and Adolescent Psychiatry, Amsterdam University Medical Center, 1100 DD Amsterdam Zuidoost, The Netherlands; Erasmus School of Social and Behavioural Sciences, Erasmus University, Rotterdam 3062 PA, The Netherlands; Leiden Institute for Brain and Cognition, Leiden University, Leiden 2300 RC, The Netherlands; Developmental and Educational Psychology, Faculty of Social and Behavioural Sciences, Leiden University, Leiden 2333 AK, The Netherlands

**Keywords:** self-concept, antisocial behavior, psychopathy, fMRI

## Abstract

Self-concept is shaped by social experiences, but it is not yet well understood how the neural and behavioral development of self-concept is influenced by a history of antisocial behavior. In this pre-registered study, we examined neural responses to self-evaluations in young adults who engaged with antisocial behavior in childhood and either desisted or persisted in antisocial behavior. A self-concept task was performed by 94 young adults (age range 18–30 years). During the task, participants with a persistent or desistent antisocial trajectory (*n* = 54) and typically developing young adults (*n* = 40) rated whether positive and negative traits in different domains (prosocial and physical) described themselves. We examined both the effects of a history of antisocial behavior as well as current heterogeneity in psychopathic traits on self-concept appraisal and its neural underpinnings. Participants endorsed more positive trait statements than negative across domains, which did not differ between antisocial-history groups. However, current psychopathic traits were negatively associated with prosocial self-concept and medial prefrontal cortex activity during self-evaluation. Together, these findings suggest that antisocial tendencies might indeed be reflected in self-concept development of young adults, specifically in the prosocial domain.

## Introduction

Self-concept, defined as the knowledge and evaluation of ourselves, is shaped by both cognitive development and social experiences (van de Schoot and Wong, 2012). Forming a positive and stable self-concept is an important developmental milestone (Crone *et al.*, 2022), which enhances our general well-being and protects us from mental health problems (Marsh *et al.*, 2001), while persistent negative and unstable evaluations often increase the risk of problem behavior and difficulties in socially adaptive functioning (Ybrandt, 2008). Together, self-appraisal (i.e. how positively or negatively we evaluate our traits) and self-concept clarity (i.e. whether self-knowledge is clearly defined, internally consistent and stable over time) comprise the cognitive parts of the self (Campbell *et al.*, 1996; Crone *et al.*, 2022).

Recent theories indicate that these cognitive aspects of the self are important for understanding different developmental trajectories of antisocial behavior. For instance, identity and self-concept development are thought to play an important role in desistance from crime and antisocial behavior (see e.g. Rocque *et al.*, 2016). More specifically, desistance from antisocial behavior is likely preceded or accompanied by identity changes to a more positive and prosocial self (Paternoster and Bushway, 2009; Paternoster *et al.*, 2016; Rocque *et al.*, 2016). While some of these changes already start in adolescence, they may be particularly prominent during early adulthood, a period characterized by continuing identity development, changes in social contexts and exploration of social roles (Arnett, 2007; Rocque *et al.*, 2016). In particular, during adolescence, the self-concept becomes increasingly complex and multifaceted, a process supported by ongoing brain maturation and the development of higher-order cognitive capacities, such as self-reflection and perspective-taking (van der Cruijsen *et al.*, 2017, 2018; Crone *et al.*, 2022). Identifying whether and in whom these cognitive changes arise is important, given that self-concept influences how people see themselves and others in social interactions and accordingly how (well) they behave in social contexts (Crone *et al.*, 2022). Therefore, we aim to examine and characterize how a history of antisocial behavior (i.e. young adults who persisted in or desisted from antisocial behavior throughout development, relative to typically developing controls) is associated with the neural correlates of positive and negative self-concept evaluations in early adulthood. However, given that the development of antisocial behavior is marked by substantial heterogeneity, we additionally use an individual difference approach using psychopathic traits to further understand the association between self-concept appraisal and antisocial experiences.

### The neural basis of self-concept

Neuroimaging methods have demonstrated to be a valuable approach to study self-evaluations by overcoming several difficulties (i.e. that self-concept is not directly observable and that self-report measures are subject to response bias) and providing the added value of identifying neurobiological mechanisms underlying behavior. The neural basis of self-concept is often studied using trait judgment paradigms, by asking participants whether and to what extent certain trait statements are descriptive of themselves. Studies using such paradigms have repeatedly shown increased activity in the ventral and rostral medial prefrontal cortex (mPFC) (Denny *et al.*, 2012; Lieberman *et al.*, 2019; Murray *et al.*, 2012) during self-evaluations, compared to other evaluations or baseline conditions. While there is indeed ample support for general neural mechanisms underlying self-representation, research increasingly supports the view that self-concept is a multidimensional construct that also involves distinct, specific knowledge structures. In particular, self-concept evaluations and their neural underpinnings may depend on the domain (e.g. physical and prosocial) and valence (i.e. positive and negative) of self-knowledge (e.g. van der Cruijsen *et al.*, 2017, 2018). Positive self-concepts, or more applicable traits, are associated with stronger activation in the anterior mPFC (aMPFC) compared to negative traits (D’Argembeau, 2013; van der Cruijsen *et al.*, 2017).

Possibly, if people with persistent antisocial tendencies are prone to more negative self-evaluations, they show attenuated mPFC activity during self-evaluations (Herpertz *et al.*, 2018; van der Aar *et al.*, 2019). Indeed, youth diagnosed with conduct disorder show reduced mPFC activity in the rostral aMPFC during functional Magnetic Resonance Imaging (fMRI) tasks that require self-reflective thoughts (Dalwani *et al.*, 2014). This attenuated activity was accompanied by aberrant default mode network (DMN) connectivity between the aMPFC and other DMN subregions important for self-related processing, such as the posterior cingulate (Dalwani *et al.*, 2014; Broulidakis *et al.*, 2016). Moreover, prior studies indicate that mPFC is often structurally or functionally impaired in antisocial populations (de Oliveira-Souza *et al.*, 2008; Raine, 2008; Boccardi *et al.*, 2011; Ermer *et al.*, 2012; Alegria *et al.*, 2016; Fairchild *et al.*, 2019). Taken together, it is important to examine whether individuals with persistent antisocial behavior (i) evaluate their traits more negatively and (ii) show less prefrontal activity during positive self-appraisals.

Self-concept develops in different social contexts. While self-traits can be evaluated globally, they often depend on respective domains, such as prosocial traits or physical characteristics (van der Cruijsen *et al.*, 2017, 2018). In previous studies, we observed that evaluating self-traits in the physical domain (e.g. looking attractive, a positive physical trait) resulted in increased activity in the lateral prefrontal cortex [e.g. dorsolateral prefrontal cortex (dlPFC)], whereas evaluations of prosocial traits (e.g. caring for others) were associated with increased medial frontal activity (van der Cruijsen *et al.*, 2017, 2018). Considering domain specificity in the relationship between antisocial behavior and self-concept may further enhance our understanding of self-concept in individuals showing persistent *vs* desisting antisocial behavior (Ostrowsky, 2010; Paternoster *et al.*, 2016). Particularly, self-concept in the domain of prosocial behavior (such as giving and helping others) is of interest, given that individuals who desist from antisocial behavior are thought to construe a more prosocial self-image prior to and during the desistance process (Paternoster *et al.*, 2016; Rocque *et al.*, 2016) than individuals who persist in antisocial behavior.

### Psychopathic traits

While differentiation between different antisocial developmental trajectories improves our understanding of general antisocial behavioral patterns over time, additionally, using individual differences approaches helps to explain heterogeneity in antisocial behavior (Garvey *et al.*, 2016). Antisocial behavior is often accompanied by high levels of psychopathic personality traits, which contribute to the emotional, interpersonal and behavioral difficulties associated with maladaptive social behavior (van Zalk and Van Zalk, 2015). Although the overall construct of psychopathy may be associated with a negative self-concept in adults (Gudjonsson and Roberts, 1983) and with structural and functional impairments of the mPFC (Ermer *et al.*, 2012; Koenigs, 2012; Fanti *et al.*, 2018; Johanson *et al.*, 2020), recent studies suggest that while the different but interrelated dimensions of psychopathy [i.e. Grandiose-Manipulative (GM), Callous-Unemotional (CU), and Impulsive-Irresponsible (II) traits] tend to co-occur in individuals, they are associated with different behavioral outcomes and physiological and neurobiological underpinnings both in adolescence and in early adulthood. In youth, CU traits have been associated with decreased mPFC activity during self-referential processing (Bontemps *et al.*, 2022). In contrast, GM traits have been associated with increased activity in the medial frontal regions, as well as in the right dlPFC in a recent electroencephalogram (EEG) study (Bontemps *et al.*, 2022). II traits have also been associated with dysfunctionality in the dlPFC during self-processing (Bontemps *et al.*, 2022), although this deficit may be more left-lateralized (Hoppenbrouwers *et al.*, 2013; Bontemps *et al.*, 2022). However, another study did not find differences between II tendencies and medial and lateral frontal regions during self-processing (Deming *et al.*, 2018).

### The current study

Taken together, we used two complementary approaches to study self-concept and antisocial tendencies. First, we used a group-based approach to identify possible behavioral and neural differences in self-concept between individuals with different developmental trajectories of antisocial behavior (persistent, desistent and control). On a behavioral level, we hypothesized that individuals showing persistent antisocial behavior would endorse fewer positive traits and more negative traits (compared to desisters and the control group) (Hypothesis 1a; Paternoster and Bushway, 2009; Paternoster *et al.*, 2016; van der Cruijsen *et al.*, 2017, 2018). Moreover, we expected that this difference would be more pronounced in the prosocial domain than in the physical domain (Hypothesis 1b, Paternoster and Bushway, 2009; Paternoster *et al.*, 2016). On a neural level, we hypothesized (Hypothesis 2a) that individuals who persisted in antisocial behavior would show less neural activity in the mPFC compared to desisters and the control group in both domains but possibly more evidently in the prosocial domain (Herpertz *et al.*, 2018; van der Aar *et al.*, 2019). Regarding the domain specificity of the effects, we expected to replicate that the contrast physical > prosocial traits would result in stronger activity in the dlPFC, whereas the contrast prosocial > physical traits was expected to result in increased mPFC activity, across all participants (Hypothesis 2b, van der Cruijsen *et al.*, 2017, 2018). Moreover, we expected that individuals who persisted in antisocial behavior would show less neural activity in the mPFC during prosocial trait evaluations, compared to physical trait evaluations.

Second, we used an individual differences approach to investigate the role of psychopathic traits in self-evaluation. We hypothesized that different psychopathic traits dimensions would be differentially associated with self-evaluations (Hypothesis 3a, see Supplementary Materials Appendix A). Further, we explored (Hypothesis 3b) (i) whether individuals with high CU traits showed decreased mPFC activity (Marsh *et al.*, 2008) and (ii) whether individuals with high GM traits showed increased or decreased mPFC and dlPFC activity when making positive self-evaluations (*vs* negative and control evaluations). Moreover, we explored the associations between II traits and neural activity during self-appraisal.

## Methods

### Participants

In the current, pre-registered study (https://osf.io/6fgbs/), we included two subsamples, comprised (i) young adults from a childhood arrestee cohort (i.e. who were arrested by the police before the age of 12), who participated in the current and previous waves of this longitudinal study (childhood arrestee sample: *N* = 54,^1^ see van de Groep *et al.*, 2022), and (ii) young adults without a history of antisocial behavior, who completed the same measures and Magnetic Resonance Imaging (MRI) protocol (control sample: *N* = 40; see van de Groep *et al.*, 2021, 2022; see Supplementary Materials Appendix A for the procedure). Participants from the childhood arrestee sample [all arrested before the age of 12 years (initial sample *N* = 364)] were followed in their early and late adolescence, into young adulthood {see Supplementary Figure S5A for an overview of the five different time points; T1 [2003–06, mean age 10.9 (s.d. = 1.4)], T2 [2004–08, mean age 11.4 (s.d. = 1.5)], T3 [2005–08, mean age 13.1 (s.d. = 1.5)], T4 [2010–12, mean age 17.6 (s.d. = 1.4)] and T5 [2019–21, mean age 25.5 (s.d. = 1.7)]}, and were classified as showing persistent or desistent antisocial developmental trajectories (*N* = 54, see van de Groep *et al.*, 2022, see also ‘Antisocial behavior’ section).

Participants were screened for fMRI contraindications, had normal (or corrected-to-normal) vision and spoke Dutch fluently. They were excluded from fMRI analyses in case they did not perform or complete the task, if the MRI data were corrupted or if they showed excessive head motion (>3 mm), resulting in a final sample of 90 participants (control sample *n* = 38 and childhood arrestee cohort *n* = 52). Analyses on behavioral results were conducted for all participants (control sample *n* = 40 and childhood arrestee cohort *n* = 54) (for descriptive data, see ).

All subjects gave written informed consent, were debriefed about the study aim after the experiment and received a financial reimbursement for their participation. The study protocol was approved by the VU University Medical Center Medical Ethical Committee (registration number 2009.268—NL28844.029.09), with local approval from the Leiden Institute for Brain and Cognition.

### Materials

#### Self-concept task

**Fig. 1.**
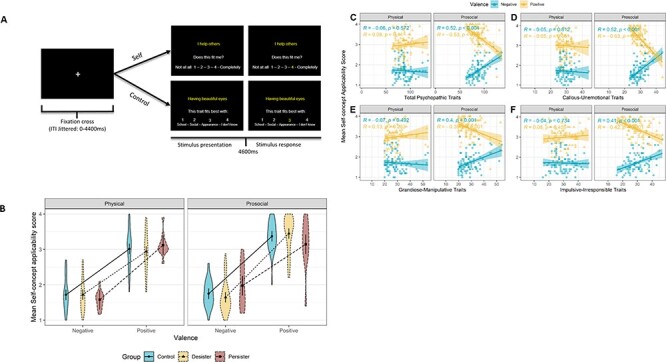
(A) Schematic depiction of the self-concept task. During the task, participants evaluate the applicability of positive and negative trait statements in two domains (prosocial and physical) on a four-point scale (self-condition), or categorize trait statements into four categories (prosocial, physical, academic and I do not know) (control condition). (B) In general, positive traits were rated as more applicable than negative traits and prosocial traits as more applicable than physical traits. (C) Correlations between positive and negative self-concept endorsement ratings and total psychopathic trait scores in the physical (left panel) and prosocial domain (right panel). (D-F) Correlations between positive and negative endorsement ratings self-concept and (sub-dimensions of) psychopathic trait scores (panel D: CU Traits, panel E: GM Traits, panel F: II Traits), in the prosocial domain and physical domain.

To investigate self-concept, participants performed an adapted version of the self-concept fMRI task (van der Cruijsen *et al.*, 2018; van de Groep *et al.*, 2021) (see Figure 1A). Note that analyses on the behavioral and neural correlates of self-concept in the control group (*n* = 40) were reported previously (van de Groep *et al.*, 2021). During the task, participants were asked to (i) evaluate whether trait statements applied to themselves (self-condition, 40 trials) on a four-point scale or (ii) categorize trait statements into four categories (prosocial, physical, academic and I do not know) (control condition, 12 trials). For both conditions, trait statements could have a positive or negative valence and be from the physical appearance or prosocial domains, with an equal distribution of valence and domains among trials.

Both task conditions (self-condition and control condition) were completed in separate runs and counterbalanced across participants. The order of trials and jitter timing were optimized for our design using Optseq2 (Dale, 1999), with jittered timing intervals varying between 0 and 4400 ms. Every trial started with a fixation cross (400 ms), followed by the display with (i) the trait description and (ii) response options (4600 ms, see Figure 1A). Upon response, the chosen response was displayed in yellow for the remaining stimulus time. If a participant did not respond in the required timeframe, a message ‘too late’ was displayed for 1000 ms.

#### Youth Psychopathic traits Inventory

Psychopathic traits were measured using the Youth Psychopathy Inventory (Andershed *et al.*, 2002). This 50-item self-report questionnaire distinguishes three trait dimensions: GM, CU and II traits. Answers are scored on a four-point Likert scale (1 = does not apply at all, to 4 = applies very well). For both subsamples (i.e. childhood arrestee cohort and control sample), the reliability of the total Youth Psychopathic traits Inventory (YPI) score and GM and II subscales was good to excellent, and the reliability of CU traits was poor (see Table 1 and van de Groep *et al.*, 2022).

**Table 1.** Sample description, group comparisons and reliability estimates

	Group			
Measure	Desister (*n* = 42)	Persister (*n *= 12)	Control (*n* = 40)	Statistical comparison
Sex (*n* males/females)	36/6	11/1	17/23	
Age [*M* (*SD*)]	26.20 (1.63)	26.62 (1.13)	22.7 (3.09)	*F*(2, 91) = 27.77, *P* < 0.001^a^
Education (*n*)				*Χ* ^2^ = 13.128, *P* = 0.04
Vocational	25	6	17	
College	9	2	18	
University	4	1	4	
Other	4	3	1	
Ethnicity				*Χ* ^2^ = 7.18, *P* = 0.71
Dutch	37	10	36	
Surinamese	0	1	2	
Turkish	1	0	0	
Moroccan	1	0	0	
Different Western	1	1	1	
Different Non-Western	2	0	1	
IQ [*M* (s.d.)]^c^	103.47 (14.13)	100.87 (11.08)	107.35 (11.52)	*F*(2, 91) = 4.12, *P* =0.009
YPI total psychopathic traits [*M* (s.d.)]^d^	84.91 (16.37)	106.67 (19.51)	87.78 (9.68)	*F*(2, 91) = 10.89, *P* < 0.001^b^
YPI CU traits [*M* (s.d.)]	28.77 (3.64)	33.5 (5.03)	29.20 (3.05)	*F*(2, 91) = 8.28, *P* < 0.001^b^
YPI GM traits [*M* (s.d.)]	27.53 (7.47)	37.33 (11.16)	28.38 (4.96)	*F*(2, 91) = 9.22, *P* < 0.001^b^
YPI II traits [*M* (s.d.)]	28.59 (7.53)	35.83 (7.19)	30.12 (5.66)	*F*(2, 91) = 5.37, *P* = 0.006^b^
Cronbach alpha	Childhood arrestee cohort		Control group	
YPI total	0.92		0.81	
YPI CU traits	0.52		0.39^e^	
YPI GM traits	0.91		0.78	
YPI II traits	0.86		0.77	

Note. IQ, estimated IQ based on two subscales of the Wechsler Adult Intelligence Scale-IV (similarities and block design), YPI = Youth Psychopathic traits Inventory.

a Significant differences between controls and desisters and between controls and persisters.

b Significant differences between persisters and desisters and between persisters and controls.

c Note that for three participants who completed the fMRI session, the IQ tests at T5 were not completed. Therefore, we estimated these scores using multiple imputation based on the other variables reported in this table, as well as prior IQ scores (T4).

d Note that for two participants who completed the fMRI session, the YPI was not completed. Therefore, we estimated these scores using multiple imputation, based on the other variables reported in this table, as well as prior IQ scores (T4).

e Note that we did use and report a measure with a reliability lower than 0.5 (for CU traits in the control group) contrary to our pre-registered plans, given the widespread use of this measure in psychopathy research. Similar to earlier reports, we note the limitation of this approach in the discussion and acknowledge that these traits are difficult to capture reliably (although we did manage to do so for the childhood arrestee cohort and total YPI scores).

#### Antisocial behavior

For the childhood arrestee cohort, we determined whether individuals met the criteria of persistent antisocial behavior (see also van de Groep *et al.*, 2022), which was true if they showed an early onset (i.e. were convicted for an index crime before the age of 12) and received a recent diagnosis of disruptive behavior disorder (DBD) or antisocial personality disorder (ASPD) (at wave 4/5 of the longitudinal study; also see Supplementary Table S3). DBD diagnoses at the previous waves were determined using the National Institute of Mental Health Diagnostic Interview Schedule for Children (Shaffer *et al.*, 2000), while ASPD at the current wave was determined by using the International Neuropsychiatric Interview (MINI) (Lecrubier *et al.*, 1997). Of the 54 participants who completed the self-concept task, 12 were classified as persister and 42 as desister (see Table 1 and Supplementary Table S3). One participant could not be classified due to incomplete MINI administration and was therefore excluded from all subgroup analyses. As can be seen in Table 1, there were significant differences in psychopathic traits (total and subscale) scores between persisters, desisters and controls, with *post hoc* tests revealing persisters scoring significantly higher on all traits compared to the other two groups: all *t*’s < −2.597 and all *P*’s < 0.029.

### Behavioral analyses

Behavioral and region of interest (ROI) data were analyzed using R (Version 4.0.1, R Core Team, 2020). Assumptions were checked for all analyses. We identified several univariate outliers (>3 s.d.) in the psychopathic traits, for total scores and sub-scores (i.e. total psychopathic traits: 1; CU traits: 1 and GM traits: 3). However, given that these extreme values were valid scores, and removing them did not change any of the reported results, we retained them in the analyses we report here.

### Neuroimaging methods

#### Neuroimaging methods: MRI data acquisition

For acquiring (functional) MRI data, we used a 3T Philips scanner (Philips Achieva TX, Erlangen, Germany) with a standard eight-channel whole-head coil. The self-concept task was projected on a screen and viewed through a mirror on the head coil. Head movement was restricted by placing foam inserts inside the coil. Functional scans were acquired during two runs of 120 (self-condition) and 40 (control condition) dynamics, using T2* echo-planar imaging. The volumes covered the entire brain [repetition time (TR) = 2.2 s; echo time (TE) = 30 ms; sequential acquisition, 38 slices; voxel size 2.75 × 2.75 × 2.75 mm; field of view (FOV) = 220 × 220 × 115 mm]. Before the first functional scan of each run, five dummy scans were acquired. Prior to the self-concept task, we collected a high-resolution three dimensional T1 scan for anatomical reference (TR = 7.6 ms, TE = 3.5 ms, 140 slices, voxel size 1.1 × 1.1 × 1.1 mm, FOV = 250 × 196 × 170 mm).

#### Neuroimaging methods: pre-processing

Data were pre-processed and analyzed using Statistical Parametric Mapping 12 (SPM12; Welcome Department of Cognitive Neurology, London, UK). Images were corrected for slice timing acquisition and rigid body motion. We spatially normalized functional volumes to T1 templates and performed spatial smoothing using a 6 mm full width at half maximum isotropic Gaussian kernel. Subsequently, all volumes were resampled to voxels of 3 mm^3^. Templates were based on the MNI305 stereotaxic space (Cocosco *et al.*, 2003). To ensure quality control, functional images were visually checked before pre-processing and following each pre-processing step.

#### Neuroimaging methods: first-level analyses

To perform statistical analyses on individual subjects’ fMRI data, we used the general linear model in SPM12. We modeled the fMRI time series as a series of zero-duration events with a canonical hemodynamic response function, using ‘Physical-Positive’, ‘Physical-Negative’, ‘Prosocial-Positive’ and ‘Prosocial-Negative’ as regressors for the self-evaluation part of the task. For the control condition, we only modeled one event-related event: ‘Control’ (i.e. not divided into separate contrasts by valence or domain). Trials with no response were modeled separately as a regressor of no interest and were excluded from analyses (0.78% of trials). Six motion parameters were included as nuisance regressors. The pairwise comparisons resulted in participant-specific contrast images, which we subsequently submitted to the second-level group analyses.

#### Neuroimaging methods: second-level analyses

To explore whole-brain neural responses to self-representation, we performed two analyses. First, to reveal regions that were specific for self-evaluations, we compared self-condition trials (collapsed across domains and valences) to control trials using a one-sample *t*-test for the contrast Self > Control and the reversed contrast. Second, to examine valence- and domain-specific neural activity, we performed a whole-brain 2 (Valence: Positive *vs* Negative) × 2 (Domain: Physical *vs* Prosocial) Analysis of variance (ANOVA). All results were corrected using a primary voxel-wise threshold of *P* < 0.001, and coordinates for local maxima are reported in Montreal Neurological Institute (MNI) space. Unthresholded statistical maps of all reported whole-brain analyses are available on NeuroVault (Gorgolewski *et al.*, 2015); see https://neurovault.org/collections/DNPFSQNK/.

#### Neuroimaging methods: ROI analyses

The *a-priori* ROIs in which we test our main hypotheses were defined anatomically and based on previous research: medial PFC (coordinates: *x* = − 6, *y* = 50, *z* = 4; based on a meta-analysis by Denny *et al.*, 2012, cf. van de Groep *et al.*, 2021) and dlPFC (coordinates left dlPFC: *x* = − 48, *y* = 35, *z* = 16; coordinates right dlPFC: *x* = 48, *y* = 35, *z* = 16, based on van der Cruijsen *et al.*, 2017). All ROIs were created by extracting 10 mm spheres around the specified coordinates. For all ROIs, we applied Bonferroni correction for correlated variables with a threshold of *α* = 0.011 (Perneger, 1998).

## Results

### Behavioral results

#### Group-based differences in domain- and valence-specific self-evaluations

A mixed-measures ANOVA [Valence (Positive *vs* Negative) × Domain (Prosocial *vs* Physical) and Group (Persister *vs* Desister *vs* Control)] on self-ratings revealed a main effect of Valence [*F*(1, 90) = 362.41, *P *< 0.001, η_p_^2^ = 0.801] and Domain [*F*(1, 90) = 42.12, *P *< 0.001, η_p_^2^ = 0.319]. Participants rated positive items and prosocial traits as more applicable (see Figure 1B). There was no significant interaction of Group × Valence [*F*(2, 90) = 0.30, *P *= 0.743, η_p_^2^ = 0.007] (disconfirming Hypothesis 1a) nor of Valence × Group × Domain [*F*(2, 90) 2.73, *P *= 0.071, η_p_^2^ = 0.057] (disconfirming Hypothesis 1b). Note that adding age, sex, Intelligence quotient (IQ) and education as covariates did not change the results (see Supplementary Materials Appendix B).

#### Individual differences in domain- and valence-specific self-evaluations

Next, we tested the pre-registered hypothesis (3a) that total score and sub-dimensions of the YPI would be (differentially) associated with the endorsement of positive and negative traits. The associations for the separate trait dimensions were all in the same direction, showing a positive relationship between the psychopathic trait scores for CU, GM and II traits and the applicability of negatively valenced self-traits and a negative relationship with the applicability of positively valenced self-traits (see Figure 1D–F, and Supplementary Materials Appendix B). Additionally, higher total psychopathic traits scores were associated with increased endorsement of negative trait statements and decreased endorsement of positive trait statements but only for evaluations in the prosocial [Negative: *r*(91) = 0.51, *P *< 0.001, Positive: *r*(91) = −0.54, *P *< 0.001] and not in the physical domain [Negative: *r*(91) = −0.06, *P *= 0.054, Positive: *r*(91) = 0.09, *P *= 0.396] (Figure 1C). In line with these observations, a mixed-measures ANOVA on average applicability scores revealed a significant interaction between Domain, Valence and YPI total scores [*F*(1, 91) = 24.58, *P *< 0.001, η_p_^2^ = 0.21].

### fMRI results

#### Self-evaluative, domain- and valence-specific neural activation

**Fig. 2.**
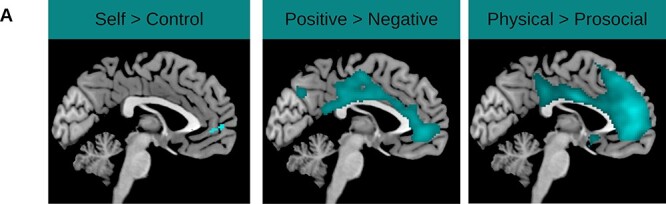
Whole-brain *t-*contrasts conducted at the group level for the contrasts: Self > Control, Positive > Negative and Physical > Prosocial.

To examine which neural activation was specific for self-evaluations, we examined the following contrasts within a whole-brain *t*-test (see Supplementary Table S4, Figure 2 and Supplementary Materials Appendix A for an overview of the results).

#### Group differences in task-, valence- and domain-specific neural activation in the mPFC

To test whether individuals who persisted in antisocial behavior showed less neural activity in the mPFC (compared to desister and control groups) (Hypothesis 2a), we conducted two mixed-measures ANOVAs using Group as between-subjects factor and Condition (for the contrast positive Self > Control) and Valence (Positive *vs* Negative) as within-subjects factors, respectively. There was no interaction effect between Group and Condition, *F*(2, 89) = 0.09, *P *= 0.918 (see Supplementary Figure S1) (disconfirming Hypothesis 2a) nor between Group and Valence, *F*(2, 89) = 1.81, *P *= 0.170, η_p_^2^ = 0.041 for the contrast Positive > Negative valence, suggesting no group differences in valence-related activity patterns of mPFC activity (see Supplementary Figure S2).

Next, we examined whether evaluating prosocial traits resulted in increased mPFC activity relative to physical traits and whether these patterns differed between groups (Hypothesis 2b-2). While there was indeed a main effect of Domain, the direction of this effect was opposite to our hypothesis, showing that evaluating physical statements evoked increased activity in the mPFC, *F*(1, 89) = 142.64, *P *< 0.001, η_p_^2^ = 0.616 (see Supplementary Figure S3C). Also contradictory to our hypotheses, there was no Group × Domain interaction (Hypothesis 2b-2 and 2b-3) [*F*(1, 89) = 0.70, *P *= 0.51]. Hence, we found no evidence (Hypothesis 2b-2) for less neural activity in the mPFC in individuals who persisted in antisocial behavior when evaluating prosocial statements, compared to when they evaluated physical trait statements, *t*(86) = 2.69, *P *= 0.088. Note that adding age, sex, IQ and education as covariates did not change the results (see Supplementary Materials Appendix B).

#### Group differences in domain-specific neural activation in the dlPFC

Our next aim was to investigate whether we could replicate prior findings showing that evaluating physical traits (*vs* prosocial traits) results in stronger activity in the dlPFC, whereas evaluating prosocial traits (*vs* physical traits) results in increased mPFC activity, across all participants (Hypothesis 2b-1). First, we conducted two mixed-measures ANOVAs using the activity extracted from the left and right dlPFC ROIs as a dependent variable and Valence, Group and Domain as independent variables. In line with Hypothesis 2b, evaluating physical traits resulted in increased activity in the left dlPFC [*F*(1, 89) = 114.20, *P *< 0.001, η_p_^2^ = 0.562; see Supplementary Figure S2] and right dlPFC [*F*(1, 89) = 12.65, *P *< 0.001, η_p_^2^ = 0.128] compared to prosocial traits (see Supplementary Figure S2). These analyses did not reveal main effects of Group or interaction effects between Group and Conditions or Valence, all *P*’s > 0.178. Note that adding age, sex, IQ and education as covariates did not render any of these effects significant.

#### Associations between the neural basis of self-concept and psychopathic traits

##### mPFC.

Our next aim was to test whether specific sub-dimensions of the YPI would be differentially associated with mPFC activity (Hypothesis 3b), using the difference scores in ROI parameter estimates between (i) Self > Control, (ii) Positive Self > Control, (iii) Positive Self > Negative Self and (iv) Prosocial > Physical. As can be seen in Figure 2B, CU traits were negatively associated with the difference score in ROI parameter estimates between Self > Control, *r*(90) = −0.28, *P* = 0.0084, suggesting that higher CU traits were associated with decreased mPFC activity during the self-condition, compared to the control condition. In line with this observation, a repeated-measures Analysis of covariance (ANCOVA) with Condition (Self *vs* Control) and CU traits as covariate revealed a significant interaction effect between Condition and CU traits, *F*(1, 86) = 7.88, *P *= 0.006, η_p_^2^ = 0.046. None of the remaining associations with YPI subscales was significant.

##### dlPFC.

Next, we explored whether specific sub-dimensions of the YPI would be differentially associated with left and right dlPFC activity (Hypothesis 3b), using the difference scores in ROI parameter estimates between (I) Self > Control, (II) Positive Self > Control, (III) Positive Self > Negative self and (IV) Prosocial > Physical. For the left dlPFC, none of the associations was significant.

**Fig. 3.**
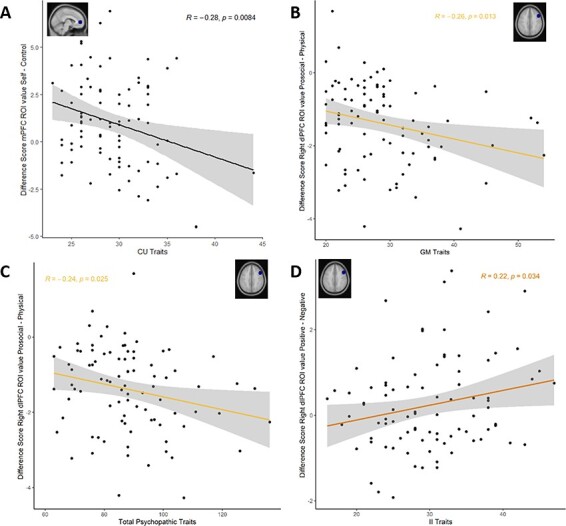
(A-D) Correlations between difference scores in mPFC and right dlPFC activity and (sub-dimensions of) psychopathic traits. (A) The mPFC activity difference score between the contrast Self > Control and CU traits was negatively correlated. (B, C) The dlPFC activity difference score between the contrast Prosocial > Physical and GM and total psychopathic traits, respectively, showed a negative, but non-significant association that did not survive Bonferroni correction (*P* < 0.11). (D) The dlPFC activity difference score between the contrast Positive > Negative and II traits was positively, but non-significantly associated, as it did not survive Bonferroni correction (*P < *0.11).

For the right dlPFC, we observed an interaction effect between GM traits and Domain, *F*(1, 86) = 5.91, *P* = 0.017, ηp^2^ = 0.009. GM traits were negatively but non-significantly associated with the difference score in ROI parameter estimates between Prosocial > Physical, *r*(90) = −0.26, *P* = 0.013 (see Figure 3B) given that this negative association did not survive Bonferroni correction (*P* < 0.11). Likewise, we observed an interaction effect between Total psychopathic traits and Domain, *F*(1, 86) = 4.53, *P* = 0.036, ηp^2^ = 0.036. Total psychopathic trait scores were negatively but non-significantly associated with the difference score in ROI parameter estimates between Prosocial > Physical, *r*(90) = −0.24, *P* = 0.025 (see Figure 3C), given that this negative association did also not survive Bonferroni correction (*P* < 0.11). Finally, II traits were positively but non-significantly associated with the difference score in ROI parameter estimates between Positive > Negative, *r*(90) = 0.22, *P* = 0.034 (see Figure 3D), given that this association likewise did not survive Bonferroni correction (*P* < 0.11). In line with this, a follow-up ANCOVA showed no significant interaction effect between II traits and Valence, *F*(1, 86) = 1.64, *P* = 0.204. In addition, none of the remaining associations with YPI sub-scores or total scores was significant.

## Discussion

In the current study, we examined whether young adults with diverging developmental trajectories of antisocial behavior differed in the endorsement of positive and negative self-evaluations and corresponding neural responses. To this end, participants completed a self-evaluation task, rating trait statements that varied in valence (positive *vs* negative) and domain (prosocial *vs* physical). To account for heterogeneity in antisocial behavior, we additionally examined the role of different sub-dimensions of psychopathic traits in self-evaluation and associated neural correlates.

Five findings stand out: first, we replicated findings from earlier studies showing that (I) people find positive self-traits more applicable than negative traits and prosocial traits more applicable than physical traits (van der Cruijsen *et al.*, 2017, 2018; van de Groep *et al.*, 2021); (II) evaluating self-traits in general, and positive traits specifically, results in increased activity in cortical midline structures, such as the mPFC (*vs* negative traits); and (III) evaluations in the physical (*vs* prosocial) domain result in increased bilateral dlPFC activity (Moran *et al.*, 2011; Denny *et al.*, 2012; van der Cruijsen *et al.*, 2017, 2018; Lieberman *et al.*, 2019). Unexpectedly, however, our results revealed no significant differences in self-evaluations between groups characterized by persistent, desistent or no prior antisocial behavior nor in the neural underpinnings of such behavior; (iv) however, we found that higher levels of total psychopathic trait scores were associated with increased endorsement of negative self-evaluations and decreased endorsement of positive self-evaluations in the prosocial but not in the physical domain; (v) finally, we found preliminary evidence that (sub-dimensions of) psychopathic traits might be differentially associated with mPFC and right dlPFC activity during self-evaluations. More specifically, CU traits were negatively associated with self-related mPFC activity, and total psychopathic traits were positively associated with domain-specific dlPFC activity, which was mainly driven by GM traits.

### Psychopathic traits and prosocial self-concept positivity

Developing a positive view of the self is an important developmental milestone, which promotes mental well-being and effective social relationships (Crone *et al.*, 2022; Ybrandt, 2008; Jankowski and Bąk, 2021). In our study, participants generally showed positive self-evaluations (Denny *et al.*, 2012; van der Cruijsen *et al.*, 2017, 2018; Lieberman *et al.*, 2019). However, contrary to our expectation, we did not observe differences in behavioral or neural responses between the persisting, desisting and control subgroups. This was surprising, given that repeated negative social interactions—which are often observed in individuals with persistent antisocial behavior (Paternoster and Bushway, 2009; Paternoster *et al.*, 2016)—are thought to shape one’s self-concept as more negative (Harter, 2012). However, we found that higher levels of psychopathic traits were associated with a more negative and less positive self-concept for prosocial but not for physical trait statements. Total and subscale levels of psychopathic traits were highest in participants with a persistent developmental trajectory (total and subscales; see Supplementary Figure S4). Our finding that people high (*vs* low) on psychopathic traits show less valence-specific differentiation in the applicability of prosocial traits fits with the idea that self-concept may be affected in some, but not all domains in people with externalizing tendencies (Kita and Inoue, 2017). Taken together, while antisocial and criminal behavior might indeed be reflected in how young adults evaluate their prosocial traits (Paternoster and Bushway, 2009; Paternoster *et al.*, 2016), individual differences in psychopathic traits better capture the complex association between antisocial histories and self-concept evaluation than group comparisons (Northoff and Tumati, 2019).

Why do individuals high on psychopathic traits have a more negative prosocial self-concept? A positive and accurate self-concept requires a balance between internal representations of the self and external input (e.g. social feedback from peers), and flexibility in the ability to focus attention toward the self facilitates self-regulation in social situations (Jankowski and Bąk, 2021). Previous research suggests that too much or too little self-focus is common in various internalizing and externalizing problems characterized by a negative self-concept (Zhao *et al.*, 2013; Philippi and Koenigs, 2014; Northoff and Tumati, 2019), such as depression (Davey and Harrison, 2022) and Attention Deficit Hyperactivity Discorder (Kita and Inoue, 2017). In depression and anxiety disorders, a negative self-concept is likely the result of too much self-focus and ruminative thoughts (Philippi and Koenigs, 2014; Northoff and Tumati, 2019). Psychopathy, on the other hand, has been associated with an extreme external focus and limited self-focus and self-reflection (Philippi and Koenigs, 2014; Doerfler *et al.*, 2021). Possibly, individuals high on psychopathic traits spend little time reflecting on their prosocial traits, which may prevent normative developmental processes that bias self-knowledge toward positivity (Jankowski and Bąk, 2021). Moreover, it should be noted that while a negative and unstable sense of self is generally thought to be maladaptive, in the case of psychopathy, it may arise and be adaptive in quickly changing social environments to facilitate goals associated with a fast life strategy (Doerfler *et al.*, 2021). Hence, future studies should further explore how self-concept valence relates to antisocial tendencies in different social contexts.

### The neural correlates of self-concept appraisal and psychopathic trait sub-dimensions

Although different psychopathy dimensions are interrelated, they often result in different outcomes and may differentially contribute to the etiology and maintenance of antisocial behavior (McCuish *et al.*, 2014; Salihovic *et al.*, 2014; van Zalk and Van Zalk, 2015; Miglin *et al.*, 2021). Hence, we expected that different psychopathic trait dimensions (CU traits, GM traits and II traits) would be differently associated with self-concept valence. While some associations we found were in the hypothesized direction, all three sub-dimensions showed similar behavioral patterns (i.e. showing positive associations with the endorsement of negative, but not positive, self-traits), which was echoed in the total psychopathic traits scores.

However, the different sub-dimensions were related to differences in neural functioning during self-appraisal. More specifically, higher CU traits were associated with decreased self-related mPFC activity (regardless of domain), which may reflect diminished self-relevance or personal value (D’Argembeau, 2013; van der Cruijsen *et al.*, 2023). This finding fits with an earlier EEG study on self-referential processing, showing attenuated mPFC activity in individuals high on CU traits (Bontemps *et al.*, 2022). Possibly, individuals with elevated CU traits have difficulty constraining their abstract self-referential schemas toward personally significant information (i.e. have less self-focused thoughts, Northoff and Tumati, 2019; Zamani *et al.*, 2022)—which results in a more negative self-concept. Alternatively, decreased personal relevance in psychopathy might arise from difficulties in identifying, describing and retrieving feelings about the self (Sifneos, 1973). Indeed, alexithymia—a condition characterized by difficulties in the experience, verbalization, identification and regulation of emotions(Larsen *et al.*, 2013), which is closely related to psychopathy (Dawel *et al.*, 2012) and specifically CU traits (Cecil *et al.*, 2018; Dadds *et al.*, 2018; Huffman and Oshri, 2022)—is possibly also associated with decreased mPFC activity during self-evaluations. Interestingly, previous studies show that alexithymia symptoms are more pronounced in youth high on CU traits with comorbid internalizing symptoms (Cecil *et al.*, 2018), while people with mere internalizing problems tend to over-constrain, rather than under-constrain, their attention toward self-thoughts (Northoff and Tumati, 2019; Davey and Harrison, 2022). In our study, we also observed comorbid internalizing problems during development, particularly in the group where CU traits were the highest (i.e. the persister group, see Supplementary Table S2). As such, future research is warranted to further explore different etiological mechanisms that give rise to a negative self-concept and its association with different types of (comorbid) psychopathology and maladjusted social behavior (Lee and Stone, 2012; Northoff and Tumati, 2019; Schettini *et al.*, 2021).

We also found that higher psychopathic traits, and particularly GM traits, were associated with increased domain-specific right dlPFC activity in the physical domain (*vs* prosocial domain). Increased dlPFC activity has often been found during trait evaluations of physical appearance (van der Cruijsen *et al.*, 2017; Moran *et al.*, 2011)* *and might reflect preferential selection, retrieval and/or maintenance of physical (rather than prosocial) information during self-appraisals (Curtis and D’Esposito, 2003; Balconi, 2013; Meshi *et al.*, 2016; van der Cruijsen *et al.*, 2017). Moreover, physical traits may require and recruit more image-based visualizations or retrieval, which has been attributed to the right dlPFC (Balconi, 2013). Alternatively, in individuals high on psychopathic traits, physical traits may be more readily available than prosocial traits and thus require more self-focused inhibition to constrain their thoughts (Lemogne *et al.*, 2009). These domain-related difficulties might be the result of disruptions in higher-order emotional processes, such as empathy and theory of mind (Brüne and Brüne-Cohrs, 2006; Bird and Viding, 2014). Future studies should examine these possibilities in more detail.

### Limitations and future directions

The current study has several limitations. First, while we managed to include a relatively large proportion of individuals who showed persistent antisocial behavior (i.e. 20% of the childhood arrestee cohort participants who completed the final time point), the size of this group was nevertheless small (see Table 1), which may have limited our statistical power to detect significant differences between groups. Moreover, low power also decreases the likelihood that positive findings are true positives (Button *et al.*, 2013), which should be considered when interpreting the results of the current study. Second, the reliability of the CU traits subscale was low, mainly in the control group, which fits with earlier reports that these traits are difficult to capture reliably (Salekin *et al.*, 2018, see also van de Groep *et al.*, 2022). Hence, results involving these traits should be interpreted with this limitation in mind. Third, contrary to our expectation, associations for the separate trait dimensions were all in the same direction, which hints at the possibility that they are not differentially associated with self-evaluations. Fourth, we found an association between self-appraisals and psychopathic traits, but no group differences, which can be partly explained by the small persister group size and observed descriptive differences between groups (e.g. in IQ). However, this discrepancy might also be related to closer conceptual and methodological (i.e. common-method variance) overlap between self-concept appraisals and self-report of psychopathic traits, relative to a diagnostic assessment of antisocial personality disorder or criminal offenses. Finally, our analyses on the associations between psychopathic traits and dlPFC activity specifically were not sufficiently powered to survive corrections for multiple testing. Hence, future research is needed to determine whether our findings would replicate in larger samples.

### Conclusion

In conclusion, this study shows that individual differences in psychopathic traits, rather than group differences in the presence of prior or continuous antisocial behavior, are important in determining how positively people appraise their prosocial self-concept. Moreover, we show preliminary evidence that individual differences in psychopathic traits are accompanied by different levels of neural activity in the mPFC and dlPFC during self-appraisal, which hints at the possibility that separable neural mechanisms underlie how people with psychopathic tendencies appraise their cognitive self-knowledge and constrain information during this process. Hence, our findings provide important starting points to understand why and how self-concept and identity play a role in desistance from crime and antisocial behavior.

## Supplementary Material

nsad016_Supp

## Data Availability

Data, study material and analysis code will be made available on DataverseNL upon publication.

## Footnotes

1 Note that demographic and clinical data did not differ between participants who completed the fMRI session (*N* = 54) and those who did not (*N* = 19, see Supplementary Table S1), except for IQ scores, which were higher in the included than the excluded group.
