# Supplementary material for: Through the looking glass: the neural basis of self-concept in young adults with antisocial trajectories
Source: Soc Cogn Affect Neurosci. 2023 Mar 17;18(1):nsad016. doi: 10.1093/scan/nsad016 (PMC10165683; doi:10.1093/scan/nsad016)
Supplement: nsad016_Supp [file nsad016_supp.zip › scan-22-205-File002.docx]

**Supplementary Materials**

**Appendix A**

**Introduction**

***The association between self-concept and psychopathic traits***

Various studies indicate that behavioral responses related to self-evaluations may also be differentially affected by psychopathic traits. For instance, Callous-Unemotional traits have been associated with low self-esteem in adolescents (aged 12-14; Fanti, 2013). On the contrary, high levels of grandiosity might cause people to have a positive self-regard, albeit one that is primarily defensive in nature – while people who score low on Grandiose-Manipulative traits may also display a positive self-concept, but one that is more secure and stable in nature (Ostrowsky, 2010). According to Baumeister et al. (1996), the former type of self-concept positivity causes people to engage in self-preserving antisocial behavior towards others who threaten or dispute their inflated self-view (i.e., in response to ego-threat). However, it should be noted that in children and adolescents (aged 9-18), maladaptive grandiosity or narcissism (e.g. characterized by a sense of entitlement, manipulative behavior to exploit others) was not consistently related to self-esteem in previous research, while adaptive narcissism (characterized by authority and self-sufficiency) was positively associated with self-esteem in children (Barry et al., 2003, 2007). Moreover, it is also possible that a combination of high grandiosity and low self-esteem (vs. high grandiosity and self-esteem) is particularly present among individuals who persistently show antisocial behavior throughout their development (Barry et al., 2003; Fanti, 2013; Fanti & Henrich, 2014) – although some have suggested that their aggressiveness stems from their intent to harm others, rather than from ego-threat (Hart et al., 2019). Given these conflicting findings, it remains unclear whether high or low levels of grandiosity and self-concept are to be expected in young adults with antisocial profiles.

Hence, on a behavioral level, we tested two competing hypotheses (Hypothesis 3a) for Grandiose-Manipulative traits: that these traits were (1) positively associated with endorsement of positive self-evaluations (Baumeister et al., 1996; Horvath & Morf, 2010), or (2) negatively associated with endorsement of negative self-evaluations (Barry et al., 2003; Fanti, 2013; Fanti & Henrich, 2014). We expected that Callous-Unemotional traits would be positively associated with endorsement of negative traits (Fanti, 2013). We explored the relationship between Impulsive-Irresponsible traits and self-concept but have no specific hypothesis about this association.

**Methods**

***Participants***

Participants were recruited from all over the Netherlands, with a primary focus on three areas where participants from the childhood arrestee cohort grew up and had been registered for a police offence prior to the age of twelve (Gelderland-Midden, Utrecht and Rotterdam-Rijnmond, cf. van Domburgh et al., 2011). Note that these areas cover different SES and included both rural and urban areas.

***Procedure***

Prior to participation, participants were informed about the study by telephone and through a digital information letter. Subjects from the control sample completed the study protocol in one session between June and September 2019. For participants in the childhood arrestee sample, data was collected across two sessions (a ‘home visit’ and scan session). However, because data collection for the childhood arrestee sample was ongoing during the outbreak of the COVID-19 pandemic, these ‘home visits’ were only conducted at participants’ homes between November 2019 and March 13th 2020 (i.e., start of the first lockdown in the Netherlands); and subsequently conducted through skype for business between March 14th 2020 and February 2021. From March 13th 2020 onwards, for the remainder of the childhood arrestee participants, IQ tests were administered during the MRI session, instead of the ‘home visit’ session (see also van de Groep et al., 2022).

In both subsamples, the aforementioned questionnaires (i.e., YPI and MINI) were administered prior to the scan session. During the scanning session, participants first received instructions about the protocol and performed practice versions of the fMRI task, and subsequently completed a scan protocol that included the self-concept fMRI task.

**Appendix B**

**Self-evaluative, domain and valence specific neural activation**

To examine which neural activation was specific for self-evaluations, we examined the following contrasts within a whole brain t-test. First, we tested the contrasts self > control and control > self. As expected, the contrast self > control revealed significant activation in the mPFC, while the reverse contrast resulted in activity in the left Insula, Occipital Gyrus and Precuneus. Next, to examine which neural activation was specific for the specified domains and valences within the self condition, we examined main and interaction contrasts within a 2 (domain) x 2 (valence) whole brain ANOVA (see Table 2 and Figure 2 for an overview of the results).

First, the contrast positive > negative resulted in increased activity in several cortical midline areas, including the mPFC and Inferior Parietal. The reverse contrast yielded increased activity in the Cerebellum and Inferior Frontal Gyrus. Second, we examined domain-specific neural activation by testing the contrasts prosocial > physical, and physical > prosocial. For the contrast physical > prosocial, we found significant activation in the dlPFC, as well as the Parietal and Temporal Inferior Gyrus. The reversed contrast resulted in activity in the Supplementary Motor Area and Superior Occipital Gyrus. Finally, the interaction between Domain x Valence showed that there was significantly more activity for positive vs negative traits in the physical domain compared to the prosocial domain in the following regions: Left lingual, Right Supplementary Motor Area and Superior Temporal Gyrus.

**Individual differences in domain and valence-specific self-evaluations: Psychopathic trait sub-dimensions**

All psychopathic trait sub-dimensions showed a similar pattern to the total psychopathic traits scores (see also section 3.1.2 and Figure 2). Accordingly, ANCOVAs on average applicability scores revealed (1) a significant interaction between Domain, Valence and Callous-Unemotional trait scores (*F*(1, 91)=22.17, *p*<.001, η_p_^2^=0.198), (2) a significant interaction between Domain, Valence and Grandiose-Manipulative trait scores (*F*(1, 91)=14.85, *p*<.001, η_p_^2^=0.142), and (3) a significant interaction between Domain, Valence and Impulsive-Irresponsible trait scores (*F*(1, 91)=14.00, *p*<.001, η_p_^2^=0.135).


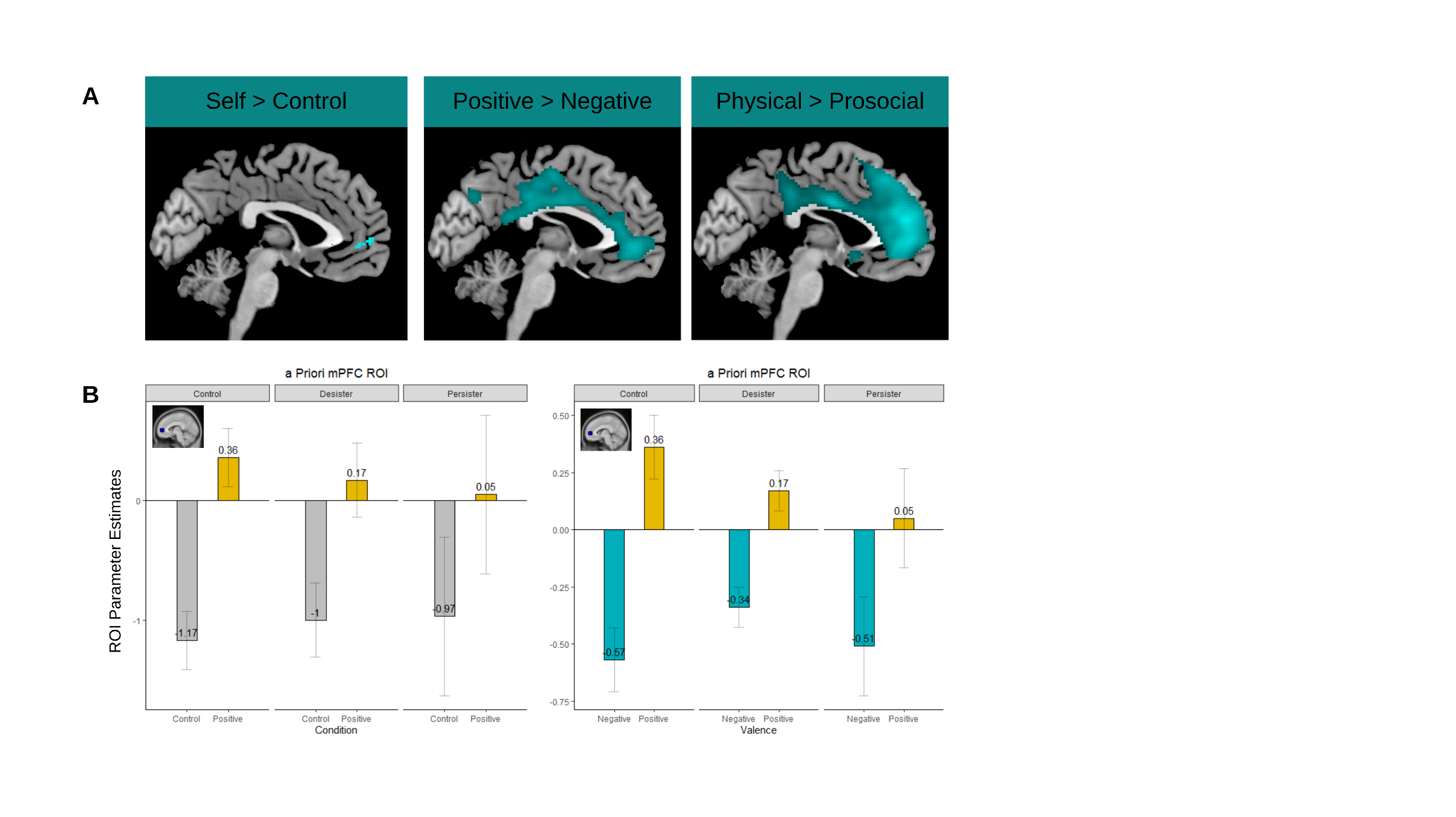


Figure S1. (A) Task condition effects for the contrast Positive Self > Control in the a prior selected mPFC based on prior meta-analyses (see methods) were similar between groups (left panel). Valence effects for the contrast Positive > Negative in the mPFC also did not differ between groups (right panel).


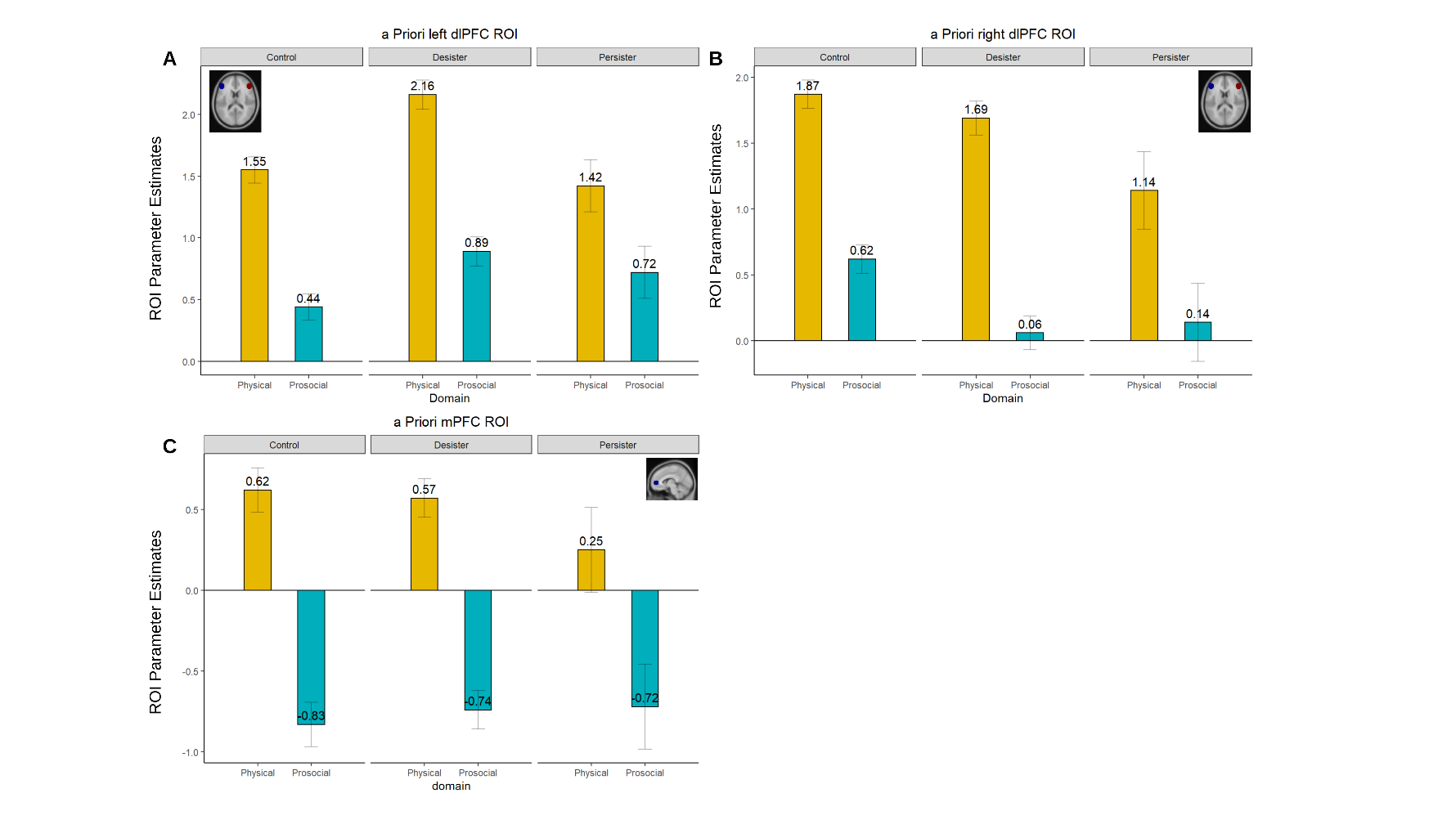


Figure S2. (A) Domain Specific effects for the contrast Physical > Prosocial in the left dlPFC (blue) were similar between groups. (B) Domain Specific effects for the contrast Physical > Prosocial in the right dlPFC (red) were similar between groups. (C) Domain Specific effects for the contrast Physical > Prosocial in the mPFC (blue) were similar between groups.


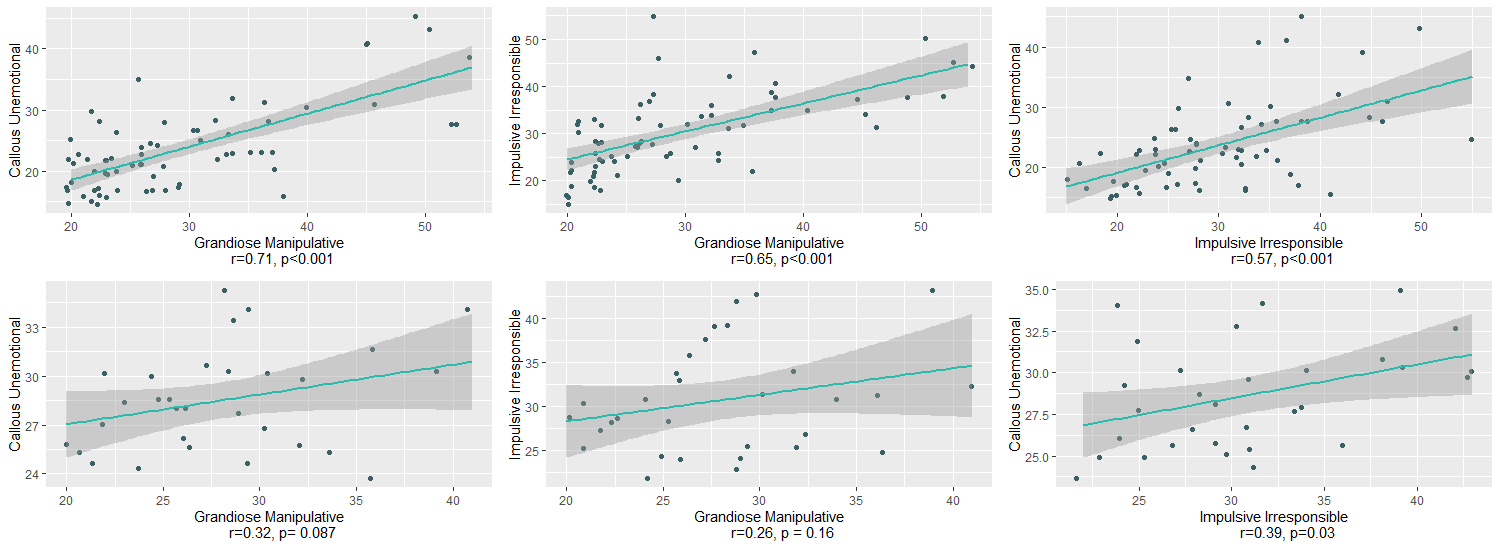


Figure S3. Correlations between the YPI subdimensions (Callous Unemotional, Grandiose Manipulative and Impulsive Irresponsible traits) in the childhood arrestee cohort (panel A) and in the control group (Panel B)


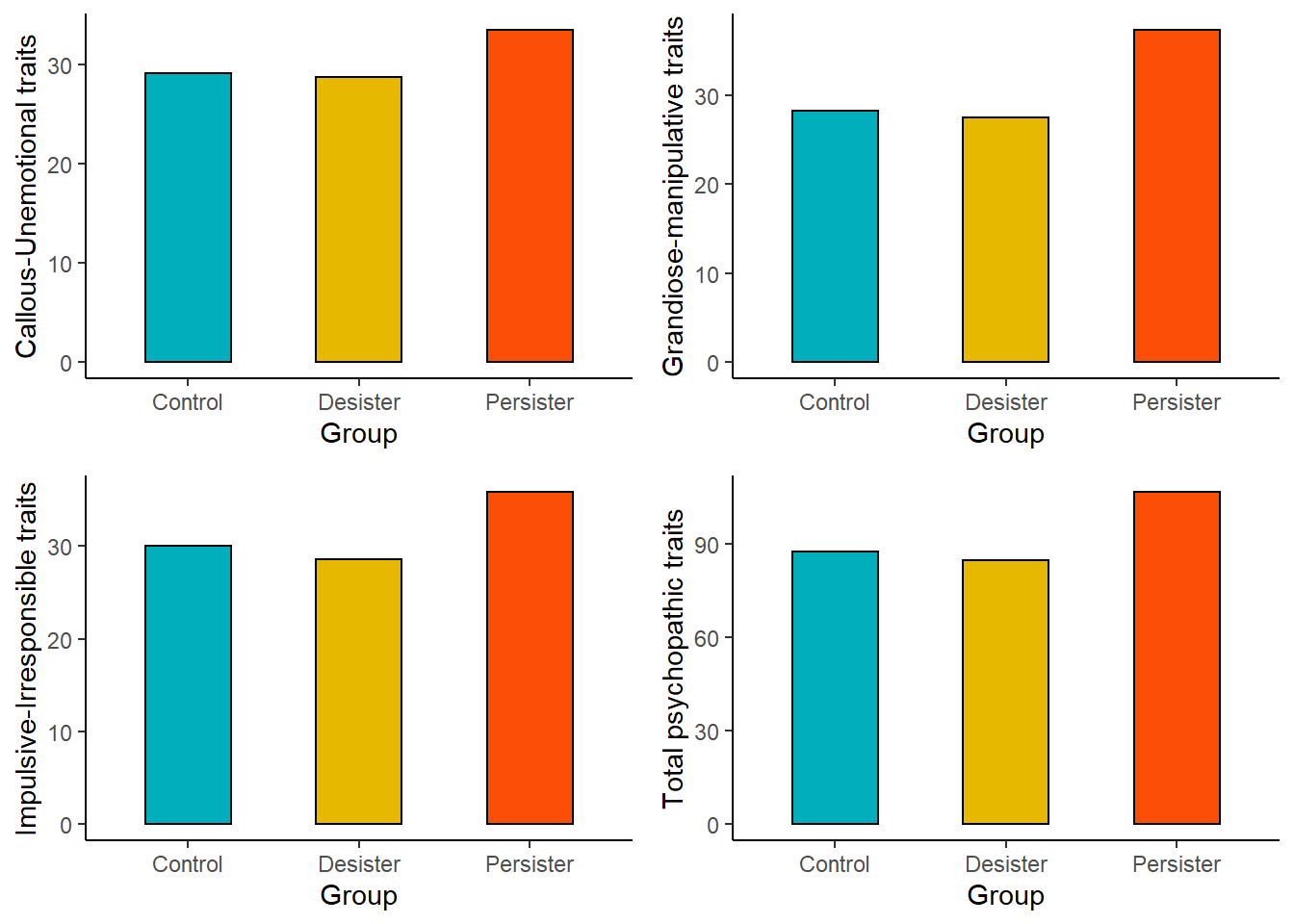


Figure S4. Mean scores for the YPI subdimensions (Callous Unemotional, Grandiose Manipulative and Impulsive Irresponsible traits) and total YPI in the Control, Desister and Persister groups.


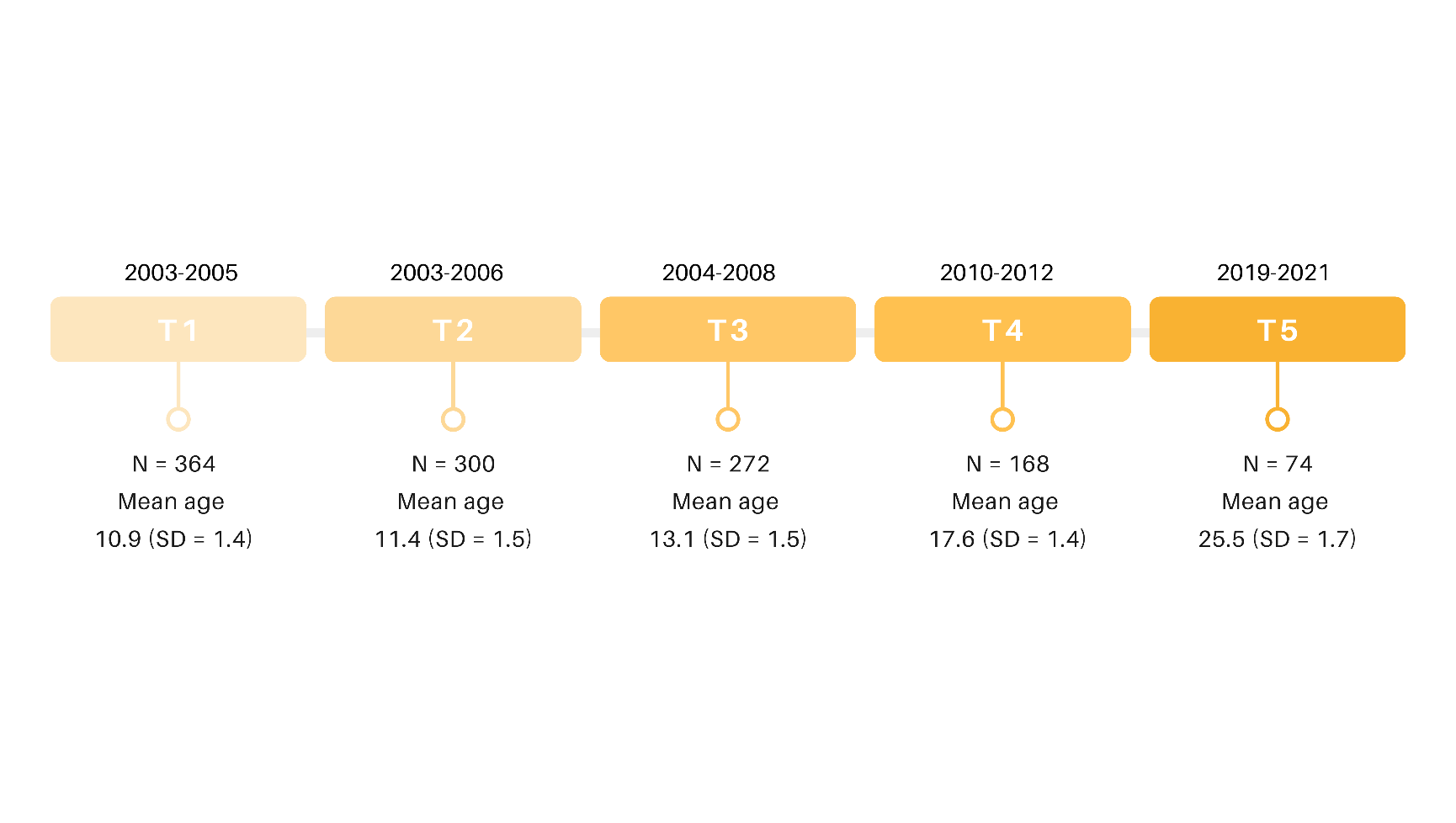


Figure S5. **(A)** Overview of the longitudinal RESIST study (Research on Individual (Anti-) Social Trajectories), aimed at investigating predictors and consequences of (anti-)social behavior across development. **(B)** Participant flow chart diagram for the fifth wave (T5) of the RESIST study.

**
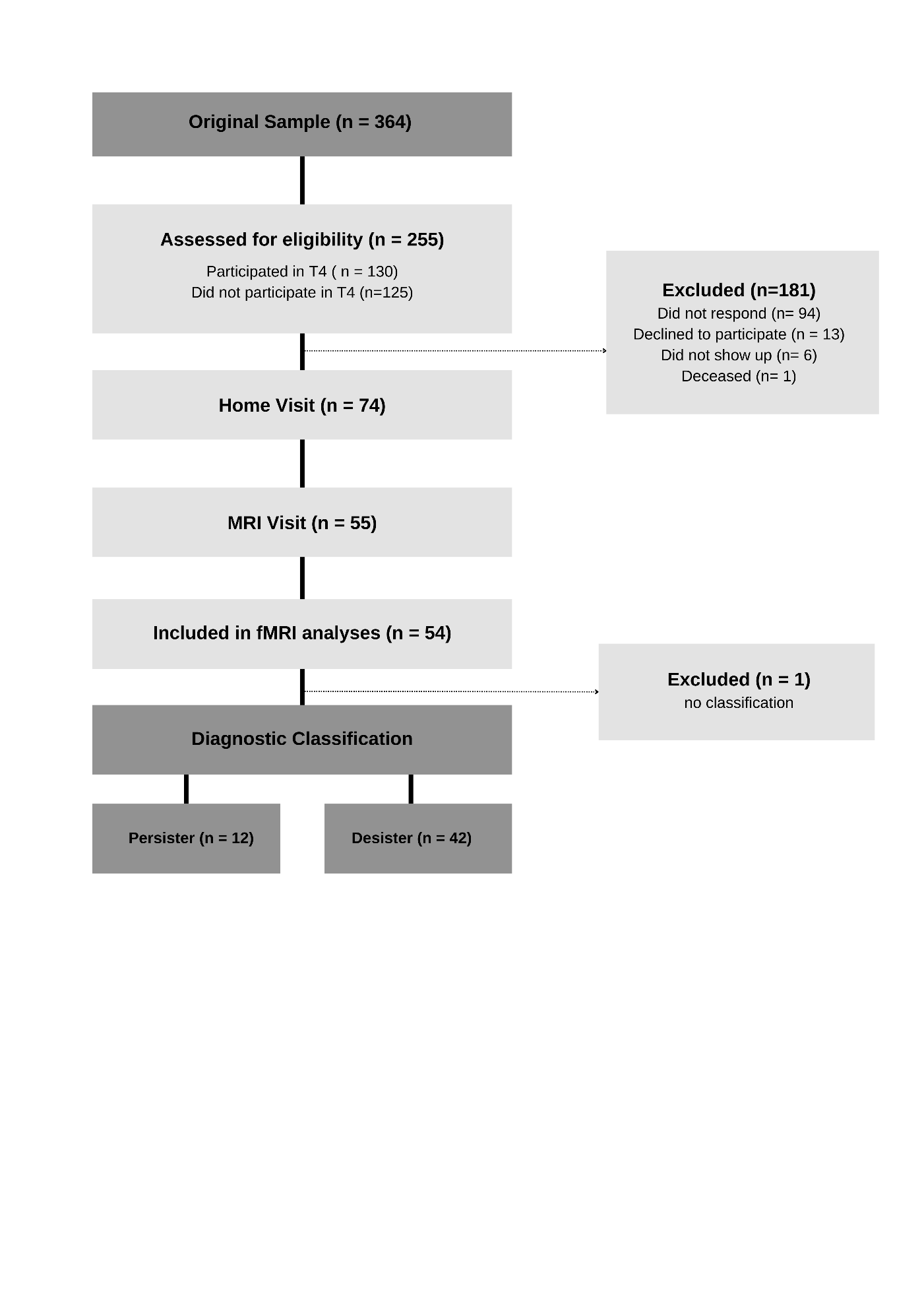
**

**Effects of Age, Sex, IQ and Education on self-evaluations**

To test whether Age, Sex, IQ and Education (i.e., the variables that differed between groups) influenced how participants in the different groups evaluated themselves, we repeated the mixed-measures ANOVA, with Valence (positive vs. negative), Domain (prosocial vs. physical), and Group (Persister vs. Desister vs. Control) as independent variables, Age, Sex and IQ and Education as covariates, and self-ratings as dependent measure.

Similar to what we reported in the main text, we observed main effect of valence, F(1, 89) = 357.09, p < .001, ηp2 = 0.800, and a main effect of domain, F(1, 89) = 44.96, p < .001, ηp2 = 0.336. There was no significant Group x Valence interaction, F(2, 89) = 0.31, p = .731, ηp2 = 0.007 – nor a significant Valence, Group and Domain interaction F(2, 89) = 2.71, p = .072, ηp2 = 0.057.

**Effects of Age, Sex, IQ and Education on mPFC activity during self-evaluations**

We repeated the three mixed measure ANOVAs using Group as between-subjects factor, and Condition (for the contrast positive self > control), Valence (positive vs. negative) or Domain (prosocial vs. physical) as within-subjects factors, with Age, Sex, IQ and Education as covariates. Once again there was no interaction effect between Group and Condition for the contrast positive > control, F(2, 89) =0.27, p=.76, nor between Group and Valence, F(2, 89)=1.87, p=.160, for the contrast positive > negative valence. The main effect of Domain remained significant, F(1, 89)= 70.74, p<.001, ηp2=0.451 (see Figure S3C), and the Group x Domain interaction remained non-significant (F(1, 89)=0.70, p=.501. Hence, accounting for the covariates did not change the mPFC activity results.

Table S1. Attrition analyses

|  | Excluded Sample  (N = 19) | Included Sample  (N = 54) | Statistics |
| --- | --- | --- | --- |
| No. Persisters | 8 | 12 | Χ^2^= 2.28, p = 0.13 |
| Age | M = 26.43, SD = 1.17 | M = 26.29, SD = 1.53 | T(71) = 0.35, p = 0.72 |
| IQ^1^ | M = 93.72, SD = 10.98 | M = 102.89, SD = 13.46 | T(71) = -2.67, p = 0.009 |
| No. Males | 15 | 46 | Χ^2^ < 0.001, p = 1 |
| YPI Callous-Unemotional Traits^2^ | M = 29.82, SD = 4.45 | M = 29.83, SD = 4.42 | T(71) = -0.003, p = 0.99 |
| YPI Grandiose-Manipulative Traits^2^ | M = 27.68, SD = 8.08 | M = 29.72, SD = 9.27 | T(71) = -0.85, p = 0.399 |
| YPI Impulsive-Irresponsible Traits^2^ | M = 32.21, SD = 9.15 | M = 30.20, SD = 7.99 | T(71) = 0.91, p = 0.367 |
| YPI total score^2^ | M = 89.72, SD = 17.51 | M = 89.74, SD = 19.23 | T(71) = -0.005, p = 0.99 |

^1^ Note that for 17 participants (n*_MRIcomplete_* = 3, n*_MRImissing_* = 14), the IQ tests at T5 were not completed, due to time constraints (n*_MRIcomplete_* = 3), or limitations imposed by the COVID-19 pandemic to complete the WAIS in person (n*_MRImissing_* = 14). Therefore, we estimated these scores using multiple imputation (mice package v3.13.0; van Buuren & Groothuis-Oudshoorn, 2011), based on the other variables reported in this table, as well as prior IQ scores (T4).

^2^ Note that for three participants (n*_MRIcomplete_* = 2, n*_MRImissing_* = 1), the YPI was not completed. Therefore, we estimated these scores using multiple imputation, based on the other variables reported in this table, as well as prior IQ scores (T4).

**Table S2**

*MINI diagnoses in the persister and desister groups^^[[1]](#footnote-1)^^*

|  | Desister (N = 42) | Persister (N = 12) |
| --- | --- | --- |
| MINI Diagnosis |  |  |
| Past Major Depressive Disorder | 6 (14.29%) | 5 (45.45%) |
| Current Mood Disorder due to physical condition | 1 (2.38%) | 0 (0.00%) |
| Past Mood Disorder due to drug use | 0 (0.00%) | 1 (9.09%) |
| Agoraphobia | 1 (2.38%) | 3 (27.27%) |
| Obsessive-Compulsive Disorder | 1 (2.38%) | 1 (9.09%) |
| Generalized Anxiety Disorder | 7 (16.6%) | 3 (27.27%) |
| Alcohol dependence / abuse | 15 (35.71%) | 5 (45.45%) |
| Drug (non-alcohol) dependence / abuse | 10 (23.8%) | 7 (63.6%) |
| Attention Deficit Hyperactivity Disorder | 0 (0.00%) | 1 (9.09%) |
| Posttraumatic Stress Disorder | 1 (2.38%) | 2 (18.18%) |

^1^ Note that screening for behavioral issues and clinical disorders was performed on the same day of the MRI for the controls (using the diagnostic interview), and for participants from the persister/desister group 0-375 days before the MRI session, with an average of 108 days.

**Table S3**

*Diagnoses, mean age and types of index crimes in the persister and desister groups (N = 54)*

|  | Timepoint | | | | | | | | | | | |
| --- | --- | --- | --- | --- | --- | --- | --- | --- | --- | --- | --- | --- |
|  | T1 | | | | T4 | | | | T5 | | | |
| ***DISC / MINI Diagnosis^1^*** | Desister | Persister | | Total | Desister | Persister | | Total | Desister | Persister | | Total |
| DBD^2^ | 9 | 3 | | 12^3^ | 0 | 4 | | 4 | - | - | | - |
| ASPD | - | - | | - | - | - | | - | 0 | 12 | | 12 |
| ADHD | 4 | 3 | | 7 | 4 | 0 | | 4 | - | - | | - |
| PTSS | - | - | | - | 1 | 0 | | 1 | - | - | | - |
| None | 29 | 6 | | 35 | 29 | 3 | | 32 | - | - | | - |
| Missing | - | - | | - | 8 | 5 | | 13 | - | - | | - |
|  |  |  | |  |  |  | |  |  |  | |  |
| ***Age*** | T0^4^ | | T1 | | T2 | | T3 | | T4 | | T5 | |
| Mean | 10.49 | | 10.9 | | 12.01 | | 13.01 | | 18.11 | | 26.52 | |
| SD | 1.43 | | 1.47 | | 1.53 | | 1.58 | | 1.31 | | 1.63 | |
| Min | 5.95 | | 6.21 | | 7.47 | | 8.37 | | 14.76 | | 21.6 | |
| Max | 11.97 | | 12.78 | | 14.38 | | 15.15 | | 20.38 | | 29.14 | |
|  |  | |  | |  | |  | |  | |  | |
|  | T0 | | | | | | | | | | | |
| ***Type of index crime*** | Desister (n = 42) | | | | Persister (n = 12) | | | | Total (n = 54) | | | |
| Arson | 7 | | | | 0 | | | | 7 | | | |
| Theft | 7 | | | | 1 | | | | 8 | | | |
| Violent crime | 4 | | | | 4 | | | | 8 | | | |
| Public nuisance | 11 | | | | 0 | | | | 11 | | | |
| Vandalism | 8 | | | | 4 | | | | 12 | | | |
| Missing | 5 | | | | 3 | | | | 8 | | | |

^1^ Note that the DISC was only administered at T2 or T3 if it was not administered at T1 ^2^ Means participant had at least a DBD diagnosis (DBD CD / ODD / CD + ODD / ADHD + OD, ADHD + CD, ADHD + OD + CD)

^3^ Note that of these 12 participants, 7 were diagnosed with DBD before the age of 12, and 5 after the age of 12 (during the current study).

^4^ Note that T0 corresponds to the timepoint at which participants were arrested.

| **Table S4** |  | | | | | |  |  |
| --- | --- | --- | --- | --- | --- | --- | --- | --- |
| MNI coordinates of local maxima activated the contrasts Self > Control, Control > Self, Positive > Negative, Negative > Positive, Physical > Prosocial and Prosocial > Physical; and the interaction between Valence and Domain. Results were calculated using a primary voxel-wise threshold of p < .001 (uncorrected). | | | | | | | | |
| Area of activation | MNI Coordinates | |  | | | Test statistic | | Cluster Size |
|  | x | y | | z |  | *T* | |  |
| *Self > Control* |  |  | |  |  |  | |  |
| SupraMarginal_L | 60 | -24 | | 38 |  | 5.02 | | 307 |
| Frontal_Inf_Tri_R | 52 | 10 | | 14 |  | 4.71 | | 103 |
| Frontal_Sup_Medial_R | -2 | 56 | | 2 |  | 4.66 | | 366 |
| Frontal_Mid_R | 22 | 4 | | 58 |  | 4.36 | | 230 |
| *Control > Self* |  |  | |  |  |  | |  |
| Insula_L | -42 | 24 | | 18 |  | 8.25 | | 1758 |
| Occipital_Mid_L | -20 | -82 | | -14 |  | 7.08 | | 3419 |
| Precuneus_R | -4 | -64 | | 40 |  | 4.55 | | 357 |
| Temporal_Inf_L | -50 | -42 | | 2 |  | 4.37 | | 374 |
| *Positive > Negative* |  |  | |  |  |  | |  |
| Parietal_Inf_L | -40 | -28 | | 56 |  | 9.66 | | 20189 |
| Cerebelum_6_R | 6 | -82 | | -8 |  | 7.31 | | 1963 |
| Temporal_Sub_R | 64 | -36 | | 20 |  | 5.92 | | 2598 |
| Frontal_Mid_R | -28 | 36 | | 42 |  | 4.86 | | 372 |
| Temporal_Inf_R | -52 | -66 | | 2 |  | 4.28 | | 292 |
| *Negative > Positive* |  |  | |  |  |  | |  |
| Cerebelum_Crus1_L | -8 | -80 | | -8 |  | 8.84 | | 679 |
| Temporal_Sub_L | -54 | -34 | | -2 |  | 4.25 | | 144 |
| Frontal_Inf_Tri_L | -52 | 24 | | 8 |  | 4.15 | | 151 |
| *Physical > Prosocial* |  |  | |  |  |  | |  |
| Frontal_Inf_Tri_L | -46 | 36 | | 10 |  | 12.82 | | 22647 |
| Temporal_Inf_L | -60 | -30 | | -8 |  | 7.67 | | 691 |
| Parietal_Inf_L | -32 | -72 | | 48 |  | 7.57 | | 1999 |
| Occipital_Mid_R | 40 | -68 | | 44 |  | 7.31 | | 1487 |
| Temporal_Inf_R | 56 | -40 | | -8 |  | 6.22 | | 510 |
| Precuneus_L | 12 | -64 | | 30 |  | 4.27 | | 405 |
| *Prosocial > Physical* |  |  | |  |  |  | |  |
| Supp_Motor_Area_R | -6 | 4 | | 18 |  | 5.14 | | 329 |
| Occipital_Sup_R | 16 | -48 | | -4 |  | 4.36 | | 229 |
| *Interaction Valence x Domain* |  |  | |  |  |  | |  |
| Lingual_L | -8 | -84 | | 0 |  | 9.77 | | 12175 |
| Supp_Motor_Area_R | -4 | 2 | | 62 |  | 9.03 | | 11129 |
| Temporal_Sup_L | -50 | -34 | | -2 |  | 6.73 | | 1287 |

Note: Names were based on the aal toolbox in SPM. For functional regions discussed throughout the paper, both the aal label and functional label (between brackets) are displayed. See <https://neurovault.org/collections/DNPFSQNK/> for a full, untresholded overview of activation.

**References**

Barry, C. T., Frick, P. J., & Killian, A. L. (2003). The relation of narcissism and self-esteem to conduct problems in children: A preliminary

investigation. *Journal of Clinical Child and Adolescent Psychology*, *32*(1), 139-152. https://doi.org/10.1207/S15374424JCCP3201_13

Barry, T. D., Thompson, A., Barry, C. T., Lochman, J. E., Adler, K., & Hill, K. (2007). The importance of narcissism in predicting proactive and

reactive aggression in moderately to highly aggressive children. Aggressive Behavior, 33(3), 185–197. <https://doi.org/10.1002/ab.20198>

Baumeister, R. F., Smart, L., & Boden, J. M. (1996). Relation of threatened egotism to violence and aggression: The dark side of high self-

esteem. *Psychological Review*, *103*(1), 5–33. <https://doi.org/10.1037/0033-295x.103.1.5>

Fanti, K. A., Demetriou, C. A., & Kimonis, E. R. (2013). Variants of callous-unemotional conduct problems in a community sample of

adolescents. *Journal of youth and adolescence*, *42*(7), 964-979. <https://doi.org/10.1007/s10964-013-9958-9>

Fanti, K. A., & Henrich, C. C. (2015). Effects of Self-Esteem and Narcissism on Bullying and Victimization During Early Adolescence. The

*Journal of Early Adolescence*, 35(1), 5–29. <https://doi.org/10.1177/0272431613519498>

Hart, W., Richardson, K., & Breeden, C. (2019). An interactive model of narcissism, self-esteem, and provocation extent on aggression.

Personality and Individual Differences, 145, 112–118. https://doi.org/10.1016/j.paid.2019.03.032

Ostrowsky, M. K. (2010). Are violent people more likely to have low self-esteem or high self-esteem?. Aggression and Violent Behavior, 15(1),

69-75. <https://doi.org/10.1016/j.avb.2009.08.004>

Van Buuren, S., & Groothuis-Oudshoorn, K. (2011). mice: Multivariate imputation by chained equations in R. *Journal of statistical software, 45*,

1-67. <https://doi.org/10.18637/jss.v045.i03>

1. Note that the Chi-Square Test revealed no significant differences between groups, Χ2 = 9.12, p = .43. [↑](#footnote-ref-1)
